# The Wonder Years: What Can Primary School Children Teach Us About Immunity to *Mycobacterium tuberculosis*?

**DOI:** 10.3389/fimmu.2018.02946

**Published:** 2018-12-13

**Authors:** James A. Seddon, Silvia S. Chiang, Hanif Esmail, Anna K. Coussens

**Affiliations:** ^1^Department of Paediatrics, Imperial College London, London, United Kingdom; ^2^Desmond Tutu TB Centre, Department of Paediatrics and Child Health, Faculty of Medicine and Health Sciences, Stellenbosch University, Cape Town, South Africa; ^3^Department of Pediatrics, Warren Alpert Medical School of Brown University, Providence, RI, United States; ^4^Center for International Health Research, Rhode Island Hospital, Providence, RI, United States; ^5^Radcliffe Department of Medicine, University of Oxford, Oxford, United Kingdom; ^6^Wellcome Centre for Infectious Diseases Research in Africa, Institute of Infectious Disease and Molecular Medicine, University of Cape Town, Cape Town, South Africa; ^7^Infection and Immunity Division, Walter and Eliza Hall Institute of Medical Research, Parkville, VIC, Australia; ^8^Division of Medical Biology, Faculty of Medicine, Dentistry and Health Sciences, University of Melbourne, Parkville, VIC, Australia; ^9^Division of Medical Microbiology, Department of Pathology, University of Cape Town, Cape Town, South Africa

**Keywords:** tuberculosis, children, adolescence, *Mycobacterium tuberculosis*, vaccination, infection, immunity, protection

## Abstract

In high burden settings, the risk of infection with *Mycobacterium tuberculosis* increases throughout childhood due to cumulative exposure. However, the risk of progressing from tuberculosis (TB) infection to disease varies by age. Young children (<5 years) have high risk of disease progression following infection. The risk falls in primary school children (5 to <10 years), but rises again during puberty. TB disease phenotype also varies by age: generally, young children have intrathoracic lymph node disease or disseminated disease, while adolescents (10 to <20 years) have adult-type pulmonary disease. TB risk also exhibits a gender difference: compared to adolescent boys, adolescent girls have an earlier rise in disease progression risk and higher TB incidence until early adulthood. Understanding why primary school children, during what we term the “Wonder Years,” have low TB risk has implications for vaccine development, therapeutic interventions, and diagnostics. To understand why this group is at low risk, we need a better comprehension of why younger children and adolescents have higher risks, and why risk varies by gender. Immunological response to *M. tuberculosis* is central to these issues. Host response at key stages in the immunopathological interaction with *M. tuberculosis* influences risk and disease phenotype. Cell numbers and function change dramatically with age and sexual maturation. Young children have poorly functioning innate cells and a Th2 skew. During the “Wonder Years,” there is a lymphocyte predominance and a Th1 skew. During puberty, neutrophils become more central to host response, and CD4+ T cells increase in number. Sex hormones (dehydroepiandrosterone, adiponectin, leptin, oestradiol, progesterone, and testosterone) profoundly affect immunity. Compared to girls, boys have a stronger Th1 profile and increased numbers of CD8+ T cells and NK cells. Girls are more Th2-skewed and elicit more enhanced inflammatory responses. Non-immunological factors (including exposure intensity, behavior, and co-infections) may impact disease. However, given the consistent patterns seen across time and geography, these factors likely are less central. Strategies to protect children and adolescents from TB may need to differ by age and sex. Further work is required to better understand the contribution of age and sex to *M. tuberculosis* immunity.

## Introduction

About a quarter of the global population (1), including nearly 70 million children and adolescents <15 years of age (2), is infected with *Mycobacterium tuberculosis*. Many infected individuals are able to contain *M. tuberculosis* without the organism ever causing pathology. However, in a subset, the intricate immunological response necessary to contain bacterial proliferation is lost. Of the nearly 70 million children and adolescents <15 years of age with TB infection, about 1 million develop TB disease each year (3). Young children, especially those <2 years of age, have an extremely high risk of developing TB disease after becoming infected. The risk then falls to a nadir in primary school children before rising during adolescence (4). In fact, the primary school years could be considered the “Wonder Years” of TB protective immunity: even when infected with *M. tuberculosis*, primary school children have the lowest risk, of any age throughout life, of progressing to TB disease.

**Figure 1:**
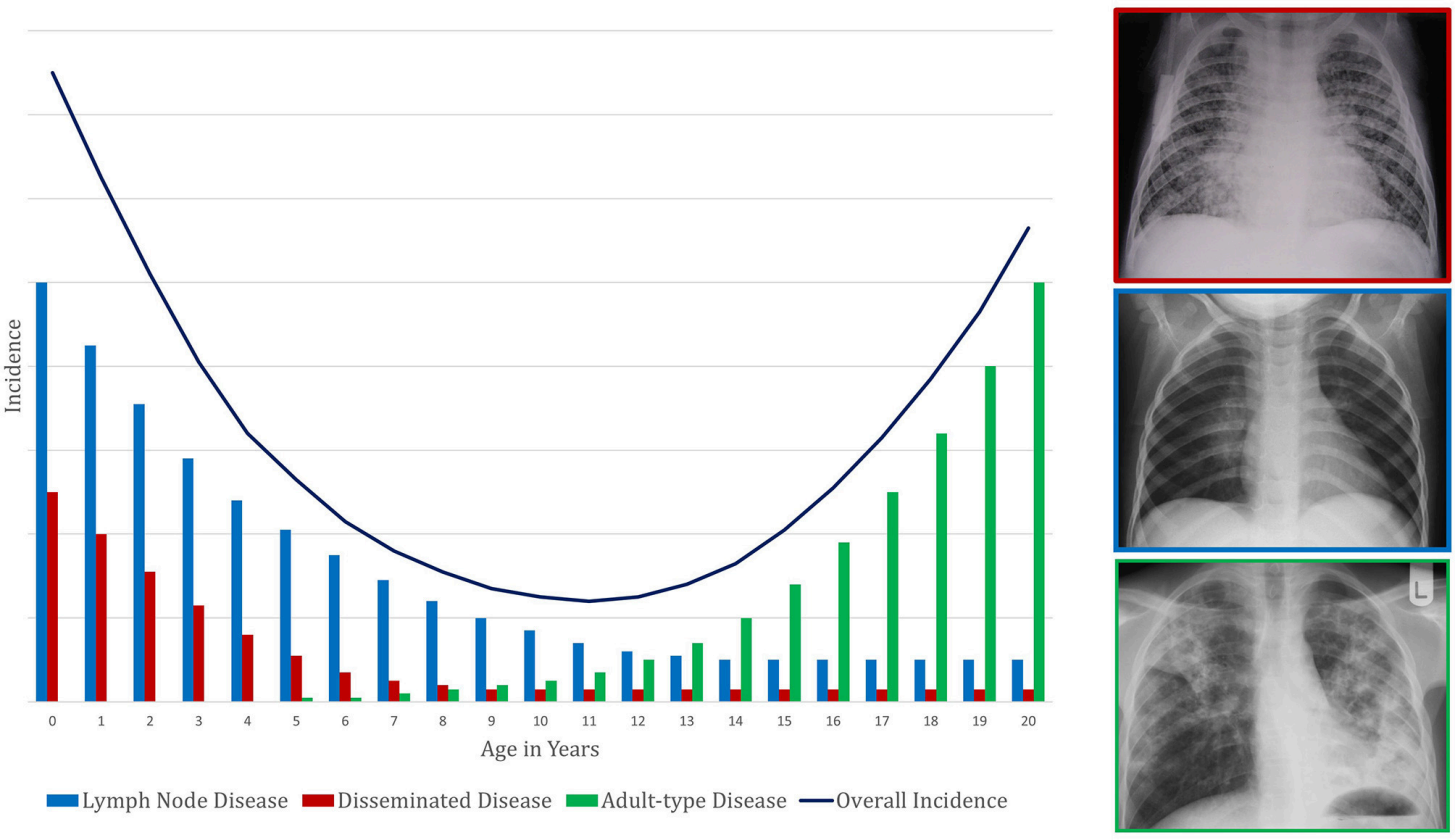
Conceptual framework to demonstrate the pattern of change in tuberculosis incidence with age. This represents a composite of risk of infection and risk of subsequent disease progression. The presentation of disease is demonstrated by a representative X-ray in a box colored according to the disease phenotype legend.

Primary school children also have the most benign clinical manifestations of TB disease: classically, this age group has paucibacillary, intra-thoracic disease with greater involvement of the mediastinal lymph nodes than the lung parenchyma. Young children (<5 years of age) also have this type of intrathoracic disease but are additionally at high risk of disseminated TB, which has a high mortality. Largely for this reason, of the nearly 250,000 individuals <15 years of age who die from TB each year, most are <5 years of age (5). Around the time of puberty, mediastinal lymph node disease and disseminated TB become uncommon. Pulmonary TB begins to present as destructive lesions of the lung parenchyma, frequently in the upper lobes and with cavitation (6–9); TB-related mortality rises again. Taken together, these observations suggest that primary school children are protected from the two extremes of TB disease: the disseminated pathology commoner in young children and the destructive pulmonary disease commoner in adolescents and adults (Figure 1). The first might be considered a failure of control of infection and the latter a failure to control disease, with primary school children possessing a balanced inflammatory response capable of both.

In this article, we present the data on age-related variations in TB progression risk and disease forms. Although no studies have directly examined the reasons for these age-based differences, we discuss possible underlying immunological mechanisms, in an attempt to garner a greater understanding of the immunological correlates of protective immunity which exist during the Wonder Years of childhood protection immunity. We will make the case that young children <5 years have deficiencies in the initial response to *M. tuberculosis* infection, which reduce the chance of successful containment of the organism. These deficiencies increase the risk of progression to disseminated disease. In contrast, adolescents develop an excessively inflammatory response in the early stages of disease, which leads to tissue damage and favors localized replication of *M. tuberculosis*. We will also highlight divergences in disease risk between adolescent boys and girls, and explore the possible impact of sex hormones on host response to *M. tuberculosis*. Finally, we discuss other potential factors underlying these age- and gender-associated differences in TB risk. Understanding why TB risk changes with age may provide insight into correlates of protection—which, in turn, may lead to the development of TB vaccines, immunomodulatory therapies, and diagnostic assays that utilize host immunological profiles.

## Definitions

It is important to define key terms that will appear throughout this article. We use “infants” to refer to children <12 months of age. For the purposes of this article, “young children” refers to those who are <5 years of age and includes infants (<1 year); “early childhood” is the corresponding time period. “Primary school children” are 5- to <10-year-olds, and “adolescents” are 10- to <20-year-olds. Early adulthood refers to the third decade of life (10). Although stages of childhood encapsulate much more than chronological age, we use these definitions because most epidemiologic data are available in 5-year age bands. As puberty varies in age of onset, these definitions do not fully capture differences in physiological stages of development. Therefore, we also employ the terms “pre-pubescent,” “pubescent,” and “post-pubescent” to differentiate children and adolescents with respect to their physical features.

Second, we apply the terms “sex” and “gender” according to their standard definitions. “Sex” refers to the physiological distinction associated with sex chromosomes, sex steroids, and reproductive organs. In contrast, “gender” comprises the social and cultural constructs of males and females, as well as one's self-identity (11). We do not consider intersex or transgender individuals in this article due to the lack of data on these groups. We have elected to use “gender” when reporting epidemiological data because of the influence of both physiology and social behavior on such measures.

Next, we differentiate “TB infection” and “TB disease.” “TB infection,” or “*M. tuberculosis* infection,” refers to an asymptomatic state evidenced only by immune sensitization to *M. tuberculosis*, as diagnosed by a tuberculin skin test (TST) or interferon gamma release assay (IGRA). These tests are limited in not being able to distinguish the timing, nor current state, of infection. The most commonly used term for this state is “latent TB infection (LTBI),” but “LTBI” is a suboptimal term because it inaccurately implies both the metabolic dormancy of the mycobacteria and its persistence in the host (12). “TB disease” refers to the wide continuum of radiologically and/or clinically apparent abnormalities caused by the host response to *M. tuberculosis*. Just as “TB infection” often is called “LTBI,” “TB disease” commonly is referred to as “active TB.”

Finally, we define pathologically distinct clinical phenotypes of TB disease (Figure 1). “Intrathoracic lymph node TB” is generally paucibacillary and results due to spread from the site of the initial mycobacterial infection (the Ghon focus) to the regional lymph nodes. This manifestation historically was encompassed within “primary TB,” a term now less commonly used. The “Ghon complex” is the name for the triad of the Ghon focus, surrounding lymphangitis, and regional intrathoracic lymphadenopathy. “Adult-type pulmonary TB” refers to disease that spreads bronchogenically within the lungs and may occur following prolonged initial control of infection. It typically initiates within the apico-posterior segments of the upper lobes or superior segment of the lower lobes; it appears as parenchymal infiltrates, often with cavitation, which facilitates contained expansion of bacillary numbers accompanying extracellular replication. Other terms for this clinical phenotype are “post-primary TB” and “reactivation TB,” now less commonly in use. While 60% of adult TB is pulmonary, localized disease can occur in other organs and is termed “extra-pulmonary” TB. “Disseminated TB,” also known as “miliary TB,” refers to the clinical manifestations of unrestrained haematogenous spread of mycobacteria (13, 14).

## Changes in Tuberculosis Disease Presentation by Age

As described above, age impacts the clinical phenotype of TB disease during childhood and adolescence (Figure 1). Pre-pubertal children, including both young children and primary school children, generally develop intrathoracic lymph node TB (15), which can have complications. If regional lymph nodes enlarge substantially, they can compress airways. If the node ulcerates into the bronchi and deposits caseous material into the airway, then inhalation will lead to dispersal of the mycobacteria throughout the portion of the lung supplied by that airway. This dispersal can lead to inflammation caused by acute hypersensitivity or segmental/lobar bronchopneumonic disease.

Young children, particularly those <2 years of age, also have a high risk of disseminated TB and/or TB meningitis. Disseminated TB can present with lung pathology (including the classic miliary picture seen on chest radiograph) and/or disseminated lesions throughout the body, including liver, spleen, gut, bone, and kidney. TB meningitis usually starts with an insidious clinical picture that rapidly progresses to neurological deficit. If untreated, both disseminated TB and TB meningitis almost universally lead to death (16, 17). During the primary school years, the risk of these disease forms is minimal. As children become older and approach puberty, they increasingly tend to develop adult-type pulmonary TB (6, 9, 18–20). This clinical phenotype also leads to disease transmission (21).

## Age-Related Risk of Tuberculosis Progression and Mortality

Our understanding of the impact of age on risk of progression from TB infection to disease comes from observational studies from the pre-chemotherapy era, many of which were summarized in a review by Marais et al. (4). This review included seven studies, which were conducted in Europe and North America after the advent of the TST and chest radiography, but prior to the discovery of anti-TB drugs and the HIV epidemic. The studies, which had a combined sample size of over 10,000 children and adolescents, evaluated the risk of progression to TB disease for TB-infected individuals of different ages. Table 1 summarizes these studies, as well as other relevant reports, including a trial of the Bacillus Calmette-Guérin (BCG) vaccine with over 82,000 TB-infected children and adolescents in the control arm (18).

**Table 1:** Age-related risks of progression to and mortality from tuberculosis disease.

**Location**	**Timeframe**	**Type of study**	**Sample size (denominator) from which risk was calculated^**a**^**	**Inclusion criteria**	**Definition/measurement of endpoint (TB disease or death)^**b**^**	**Risk per 1,000 person-years**			
						**Ages 0 to <5**	**Ages 5 to <10**	**Ages 10 to <15**	**Age 15 to <20**
**STUDIES REPORTING RISK OF PROGRESSION TO TB DISEASE, STRATIFIED BY AGE (IN YEARS) AT TIME OF DIAGNOSIS OF TB INFECTION**									
Minneapolis, MN, U.S.A. (22, 23, 24)	1921–1941	Cohort study	3,612	TST positivity at enrollment or TST-conversion during follow-up; TST positivity defined as induration of ≥5 mm to 0.1 or 1.0 mg of old tuberculin	“Clinical TB,” not including “primary pulmonary infiltrates” on CXR in the absence of symptoms	257 (age 0 to <1); 160 (age 1 to <2); 143 (age 2 to <3); 50 (age 3 to <4); 46 (age 4 to <5)^b^	48 (age 5 to <6); 38 (age 6 to <7); 45 (age 7 to <8); 78 (age 8 to <9); 49 (age 9 to <10)^b^	49 (age 10 to <11); 40 (age 11 to <12);59 (age 12 to <13); 77 (age 13 to <14); 82 (age 14 to <15)^b^	50 (age 15 to <16); 108 (age 16 to <17); 52 (age 17 to <18)^b^
Massachusetts, U.S.A. (25)	1924–1934; f/u period 1–12 (mean 11.4) years	Cohort study	64,834	Positive reaction (not further defined) to the von Pirquet tuberculin test	(1) All cases and deaths that were reported in Massachusetts from 1924 to 1936 and matched one of the individuals in the cohort, or (2) radiographic and clinical diagnosis of TB in a subset with follow-up exams	n/a	0.4	1.7	3.7
London, U.K. (26)	1930–1954; f/u period 2–25 (mean 9) years	Cohort study	1,567	History of contact with TB case; no TST required for study entry	Children developing tuberculous lesions, further divided into intrathoracic and extrathoracic	608 ^b^	432 ^b^	409 ^b^	n/a
Kinn Administrative District, Norway (27)	1937–1944; f/u period through 1945	Survey	152	≥3 mm induration to the von Pirquet tuberculin test	TB disease diagnosed through clinical symptoms, signs, and radiology	778 (age 0 to <7)	778 (age 0 to <7); 603 (age 7 to <15)	603 (age 7 to <15)	583 (age 15 to <20)
Newcastle-upon Tyne-and Northumberland, U.K. (28)	1941–1961; f/u period 1–10 years	Cohort study and literature review	2,376	8 different studies included; each study used slightly different entry criteria	Diagnoses of TB meningitis, miliary TB, pleural TB, skeletal TB, or pulmonary TB	447 (0 to <1); 48 (0 to <2); 68 (0 to <5); 265 (0 to <7)^b^	265 (0 to <7)^b^	n/a	n/a
Brentwood, Essex, U.K. (29)	1942–1953; f/u period 5–10 years	Cohort study	317	Diagnosis of simple primary TB	Complications, including pulmonary TB, grave haematogenous TB, and other extrapulmonary disease	150 (age <2); 139 (age 2 to <5)^b^	144^b^	177^b^	n/a
Puerto Rico, U.S.A. (18)	1949–1969; f/u period 18–20 years	Control arm of BCG vaccine trial	82,269	No receipt of BCG vaccine and ≥6 mm induration to 1 or 10 units of PPD	TB disease confirmed by death certificates, case reports, and reports of admission to TB hospitals and clinics	1.648 (age 1 to <7)	1.648 (age 1 to <7); 0.77 (age 7 to <13)	0.77 (age 7 to <13); 0.946 (age 13 to <19)	0.946 (age 13 to <19)
**STUDIES REPORTING RISK OF MORTALITY FROM TB DISEASE, STRATIFIED BY AGE (IN YEARS) AT TIME OF DEATH**									
Philadelphia, PA, U.S.A. (30)	1920	Epidemiologic survey	Not specified	All white children in Philadelphia (including children without TB disease)	Death from all forms of TB, data source not specified	0.33 (age <1), 0.14 (age 1 to <5)^c^	0.07^c^	0.138^c^	0.763^c^
Baltimore, MD, U.S.A. (31)	1928–1937; f/u period 1–10 years	Cohort study	1,117	Child contacts of TB cases (including children without TB disease)	Mortality from TB, data source not specified	Caucasians: 11.86 (age <1), 4.3 (age 1 to <5); African-Americans: 59.68 (age <1), 18.55 (age 1 to <5)	Caucasians: 0.88; African-Americans: 3.8	Caucasians: 0; African-Americans: 2.77	Caucasians: 7.54; African-Americans: 21.62
Massachusetts, U.S.A. (32)	1930	Epidemiologic survey	Not specified	All children in Massachusetts (including children without TB disease)	Deaths from all forms of TB, from U.S. Mortality Statistics	Males: 0.41; Females: 0.27	Males: 0.11; Females: 0.13	Males: 0.21; Females: 0.37	
Kingsport, TN, U.S.A. (33)	1930–1931	Epidemiologic survey	Not specified	All children (including healthy children) from 132 African-American households with TB	TB-related deaths reported from interviews with family members	2.9 (age <1), 0.8 (age 1 to <5)^b^	1.6^b^	4.2^b^	
Stockholm, Sweden (34)	1930–1938	Cohort study	453	Diagnosis of primary TB	Death, data source not specified	359 (age <1); 156 (age 1 to <3); 44 (age 3 to <7)^c^	44 (age 3 to <7); 8 (age 7 to <16)^c^	8 (age 7 to <16) ^c^	n/a
New York, NY, U.S.A. (35)	1930–1947	Cohort study	964	Radiologic evidence of primary TB	Deaths from TB meningitis and other complications of primary TB, including disseminated forms and local progression of primary forms	475 (age <0.5); 360 (age 0.5 to <1); 230 (age 1 to <2); 280 (age 2 to <3); 150 (age 3 to <5)^b^, ^c^	150^b^, ^c^	210^b^, ^c^	n/a
Brentwood, Essex, U.K. (29)	1942–1953; f/u period 5-10 years	Cohort study	712	Diagnosis of TB disease of any severity, including simple primary TB	Deaths from TB, data source not specified	60 (age <2); 1 (age 2 to <5)^b^	1^b^	40^b^	n/a

a *Whereas, Marais and colleagues reported the entire study population in their 2004 review article, we are reporting the population from which the risk was calculated*.

b *Risks given as per 1,000 persons rather than per 1,000 person-years*.

c *These risks were extrapolated from a line graph in the original report, and age was defined at time of diagnosis of TB disease*.

*BCG, Bacillus Calmette-Guérin; CXR, chest radiography; f/u, follow-up; PPD, purified protein derivative; TB, tuberculosis; TST, tuberculin skin test*.

The risks of disease progression described in these studies should be interpreted with caution. First, to define the age of acquisition of TB infection, some studies used baseline TST positivity and/or identification of the child or adolescent as a contact of a TB case (Table 1). Using these methods, it is difficult to establish when an individual was infected. Even if a source case is identified in the household and the child or adolescent screened soon thereafter, it is often unclear how long he or she had been exposed. It may also be unclear if the child or adolescent had prior exposure to another infectious TB case. Second, the TST has limited sensitivity and specificity to diagnose TB infection in young children. Third, the diagnosis of TB disease in children can be challenging. A clinical diagnosis can lack specificity due to the overlap in symptoms between TB and other conditions in children. Microbiological confirmation lacks sensitivity due to the paucibacillary nature of most forms of childhood TB disease, as well as the challenges in obtaining respiratory specimens from children. Finally, these studies were conducted in different settings, including wartime conditions, which likely impacted rates of progression to TB disease.

The widely varying risks of TB disease progression described in these studies reflect the limitations described above. Other reasons for the wide variation include the different quantities and varieties of tuberculin used across studies, as well as inconsistent definitions of TST positivity and TB disease. Additionally, the studies calculate risk of disease progression over different follow-up periods. Because risk of disease progression is greatest in the first year after acquisition of *M. tuberculosis* infection and then declines over time (36, 37), risks that are given per person-year inversely correlate with the length of follow-up. Not all the studies cited in Table 1 include risk of disease progression over the full spectrum of ages from infancy through late adolescence, and the studies use different age groupings. Nonetheless, as Table 1 illustrates, there is a general pattern of high risk during early childhood (particularly the first 2 years of life), a nadir during the primary school years or early adolescence (most likely occurring right before the onset of puberty), and a second peak during late adolescence. This overall picture reflects the different types of childhood TB, with falling rates of disseminated and lymph node TB in early childhood, superimposed on a rise in adult-type TB coinciding with puberty onset. Given that, in high burden settings, the risk of being infected with *M. tuberculosis* increases throughout childhood due to cumulative exposure (38), the resulting TB disease incidence seen in a community is a composite of risk of infection combined with risk of disease progression following infection. The TB incidence in high burden settings reflects the risk of disease progression, namely a U-shaped pattern, only shifted a few years older so that the nadir is at about the age of 10 years (Figure 1).

The data in Table 1 show that mortality from TB disease follows the same pattern of age-related risk. Again, despite this consistent pattern, the rates themselves vary considerably, reflecting the different conditions in which the studies were conducted and the different denominators (e.g., whole populations of geographic areas vs. children and adolescents hospitalized for TB). A recent systematic review and meta-analysis that evaluated the risk of death in children and adolescents <15 years of age with TB disease found that mortality from untreated TB disease (in the pre-chemotherapy era) was 44% in children <5 years but only 15% in 5 to <15-year-olds. Mortality in individuals with TB who were diagnosed and treated was <1% (39).

## Differences in Tuberculosis Risk by Gender

Although this article focuses on age-related differences in TB risk, it is worth noting that multiple studies have documented gender-based differences in TB risk, emerging coincident with the adolescent rise in TB risk and persists during adulthood. Overall, more males globally develop TB each year; compared to males, females tend to have higher risks of progression from TB infection to disease during adolescence. In a cohort of over 400,000 school children aged 6–19 years in Massachusetts, U.S.A., the incidence of TB disease among female TST reactors was approximately double that of male reactors (25). A TB vaccine trial that followed over 54,000 14- and 15-year-olds reported a 20% higher incidence of TB disease for girls than for boys (40). In a cohort of TB-infected children treated at Bellevue Hospital in New York City, twice as many girls as boys developed pulmonary infiltrates, and in more than a quarter of cases, adult-type pulmonary TB developed within a year of menarche (41). A study of over 82,000 children and adolescents in Puerto Rico, a trial of isoniazid prophylaxis in Alaskan Inuits, and an observational cohort in Ontario, Canada, all found that compared to males, females had higher rates of progression to TB disease in adolescence and early adulthood (18, 42, 43).

Similar patterns have been observed with respect to mortality. From 1880 to 1930 in Massachusetts, girls between the ages of 10 and 20 years had nearly twice the risk of death from TB compared to boys in the same age range (32). More recent surveys conducted in India and China also showed that females during adolescence had higher rates of mortality due to TB than males (44).

It is difficult to establish whether these data reflect a greater risk of disease progression in post-pubertal females compared to post-pubertal males on an individual level, or whether females as a group have a greater risk of disease progression because they enter puberty sooner and, thus, have longer post-pubertal periods during adolescence.

## Key Stages in the Immunopathology of Tuberculosis

**Figure 2:**
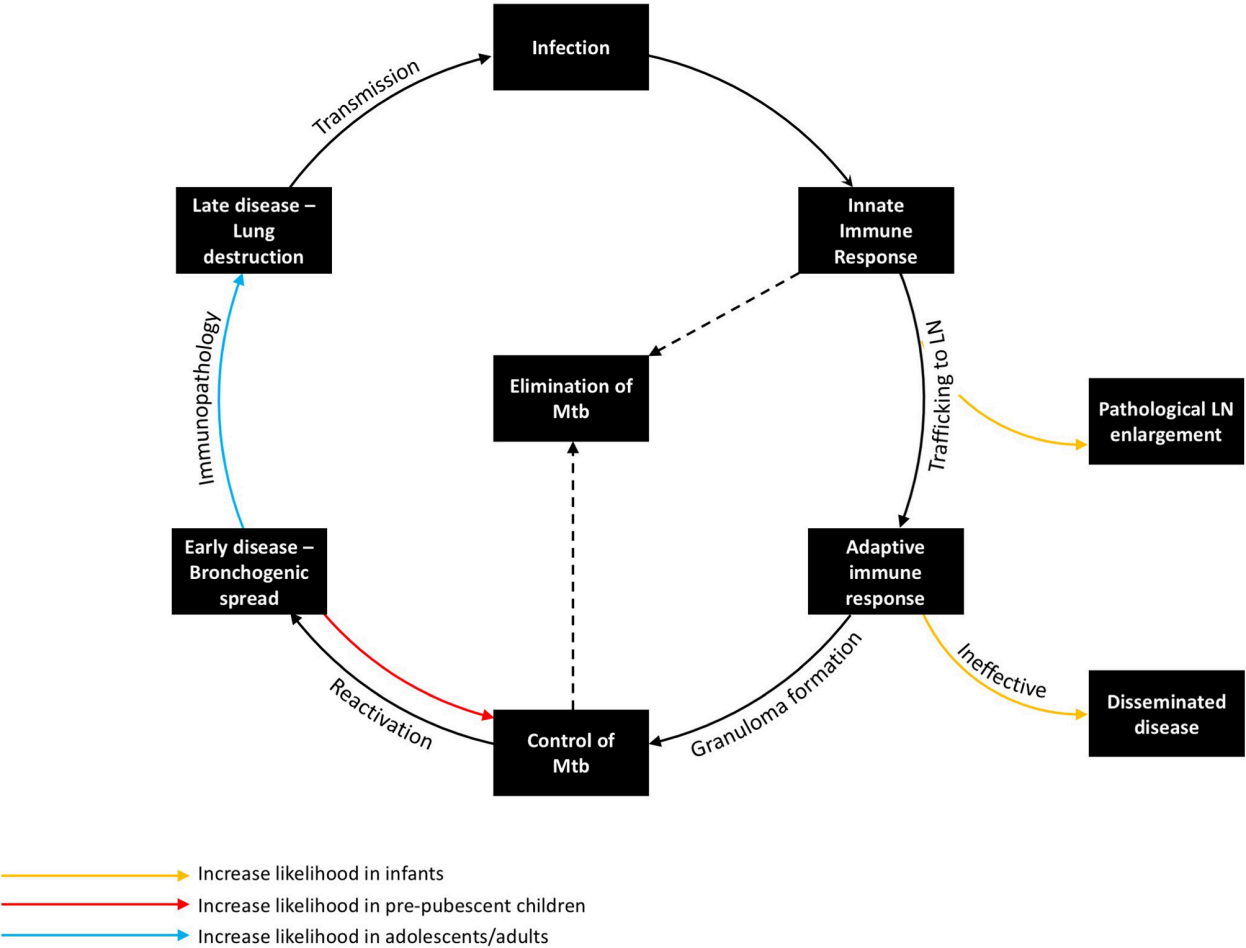
The Immunopathology of tuberculosis, demonstrating the host response at key stages in the host pathogen interaction and how age influences risk and disease phenotype. Transmission from an infectious case can lead to infection, which in turn encounters the innate immune response, the adaptive immune response, and, if not controlled, progresses to early and then late stage disease. The organism can be eliminated by either the innate or adaptive immune response. Young children are at increased risk of pathological lymph node enlargement and disseminated disease, whereas adolescents/adults are at increased risk of immunopathology. Pre-pubertal children are the most likely to contain *M. tuberculosis*.

There are a number of key stages in the host response to *M. tuberculosis* in humans, each governed by particular immune mechanisms, many of which remain incompletely understood. Age-related differences in risk and presentation may be explained by differences in the immune response at each of these key stages (Figure 2).

Exposure to infectious cases of TB can lead to inhalation of droplet nuclei containing viable *M. tuberculosis*. These infectious droplets may be cleared by the physical structures of the lung or by the innate immune system, but if they overcome these primary barriers and sensitize the adaptive component of the immune system without effective killing, the individual becomes infected with *M. tuberculosis*. Upon initial inhalation, bacilli are phagocytosed by the alveolar macrophage, which recruits neutrophils and other innate responders as a first line of defense. However, the ability of the innate immune response to control infection may be inhibited by bacterial-mediated mechanisms, particularly inhibition of phagolysosome fusion (the key bacterial killing mechanism of these phagocytes), resulting in persistence of bacilli intracellularly. It is during this period that initial antigen trafficking to the lymph nodes by dendritic cells (DCs) is felt to occur. The acquired immune response to *M. tuberculosis* usually develops 1–3 months after initial infection (as evidenced by TST or IGRA immune sensitization), with antigen-specific lymphocytes trafficking back into the lung, facilitating activation of macrophages and granuloma formation (45). At the center of the granuloma, macrophages may fuse together, forming multinucleated giant cells or differentiating into lipid-rich foam cells (46, 47). Meanwhile, the neutrophils, which are short lived, undergo necrosis, contributing to a caseous center. This granulomatous response, if able to activate macrophages sufficiently to control bacterial replication, is thought to aid in containment of bacterial spread and reduce bacillary numbers, hence controlling or potentially eliminating *M. tuberculosis*.

Deficiencies in the antigen-specific Th1 immune response, which have been well-described in children <2 years-old, likely contribute to the presentation of locally progressive and disseminated TB in this age group (48, 49). BCG vaccination boosts mycobacterium-specific cell mediated immunity, which is associated with a reduced incidence of disseminated disease (50). Following early childhood, the initial granulomatous control of infection likely is effective (51). If infection is not eliminated by this stage, there is a risk of reactivation. Factors impeding effective T-cell-macrophage interaction within the granuloma increase the likelihood of disease progression. Some of these factors are well-known causes of systemic immunosuppression, such as HIV, malnutrition, and anti-tumor necrosis factor (TNF) therapy (12). While many of the factors that precipitate reactivation are poorly understood, seasonal changes in vitamin D levels or concurrent viral infection (e.g., influenza) may be involved (52, 53). Following failure of granulomatous control of *M. tuberculosis*, a pathologically distinct phase ensues in the lung. During this phase, which typically is characterized by a pneumonic process with bronchogenic spread, the number of organisms initially is low, although abundant antigen may be detected in uninfected cells and tissue (54). Progression of this early stage of reactivation TB is not linear; regression and self-healing of lesions are common (55). A critical event during this phase appears to be lung necrosis, which is likely caused by a number of pro-inflammatory mechanisms. Following lung necrosis, an increase in bacillary number is observed (55). Recently, necrotic cells themselves have been shown to be a niche for bacillary replication (56). Contributors to this inflammatory pathology may include immune complex deposition; complement activation; neutrophil recruitment; cell-mediated cytotoxicity directed against infected and uninfected antigen-containing cells; and tissue-degrading enzymes, which have the role of facilitating cell recruitment and vascularization, but result in the breakdown of tissue architecture (57–59). The late stage of adult-type disease is then characterized by lung destruction, cavitation, and a localized exponential growth of extracellular organisms that is most commonly seen in immunocompetent adults, who are the main contributors to TB transmission (21). The lower rate of disease seen in primary school children may relate to increased likelihood of a favorable outcome during the early stage of reactivation TB, resulting in its resolution (Figure 2). It is possible that changes in the immune response that occur during puberty increase the likelihood of necrosis and disease progression.

## Impact of Age on the Host Response to *M. tuberculosis*

To understand the potential mediators of protective immunity which exists during the pre-pubertal “Wonder Years,” this unique age needs to be studied relative to the changes that occur as the immune system develops in infants and young children, and the further changes that occur during puberty. The primary driver of these developmental changes are sex hormones. Whilst the primary role of these hormones is development of reproductive organ function, they have wide-ranging effects on the immune system [for a detailed review see (60–62)]. Sex steroid receptors are found in the cytoplasm of the majority of immune cells, including T cells, B cells, DCs, natural killer (NK) cells, neutrophils, and macrophages (63). Once internalized in the cell, sex hormones bind their respective steroid receptors, inducing translocation to the nucleus and regulation of gene transcription via a variety of mechanisms. These mechanisms include direct binding to hormone response elements in DNA promoters to activate transcription; complexing with other transcription factors, such as AP-1, SP1, C/EBPβ, and NFκB; or indirectly binding DNA via chromatin-modifying co-regulators (64, 65).

**Figure 3:**
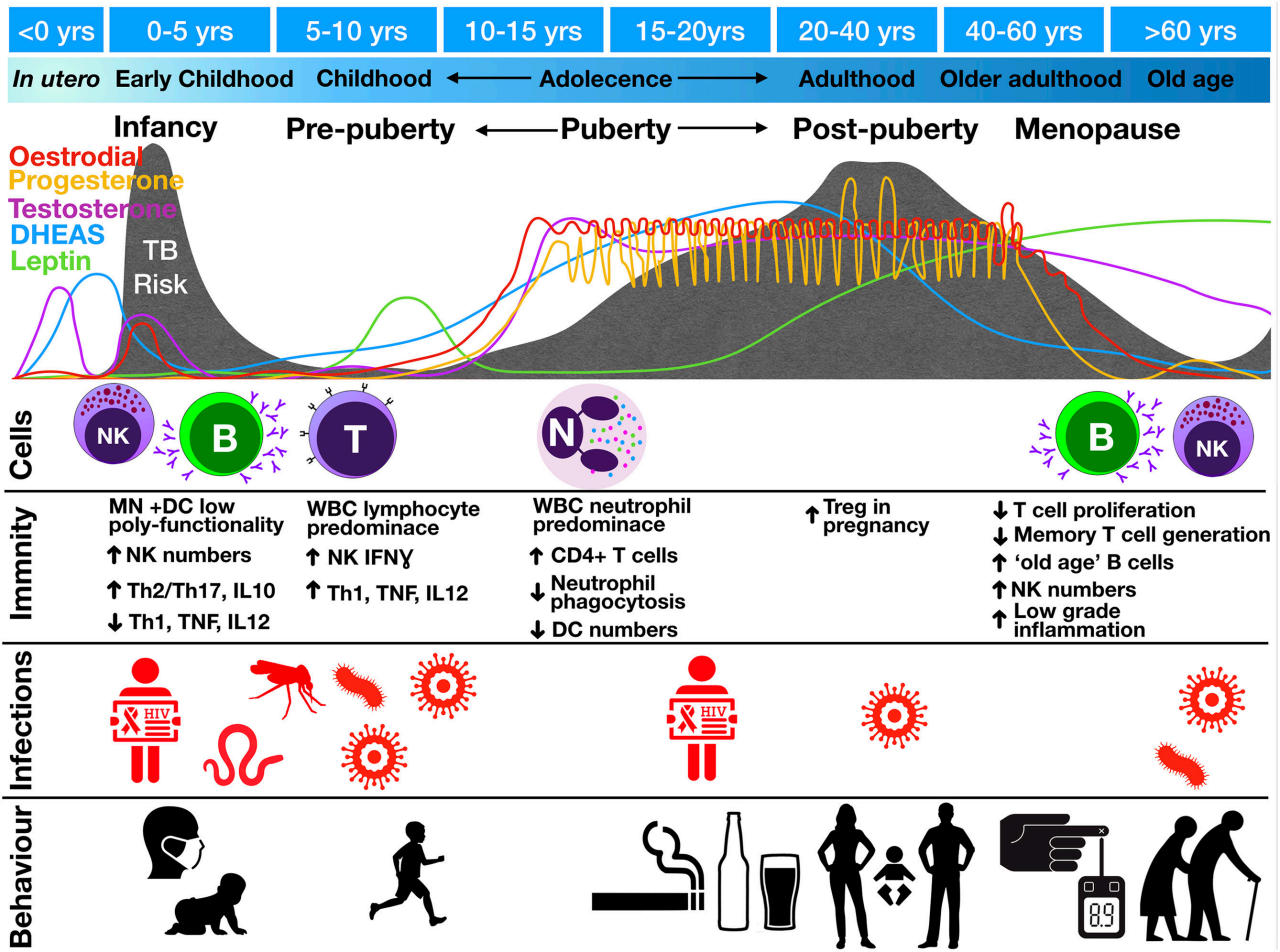
Change in tuberculosis risk throughout life stages compared to fluctuations in hormone levels, circulating cell populations (“Cells”), immune functions (“Immunity”), co-infections (“Infections”), and social interactions and behaviors (“Behavior”). TB risk is indicated by the gray shading with peaks of risk indicated underneath various life course stages. Images represent infections: mosquito, malaria; worm, helminths; virus, EBV, CMV, influenza; and behaviors: infant exposures to adult TB, children playing outdoors, smoking, drinking, sexual activity, pregnancy, Diabetes Mellites and old age. B, B cells; DC, dendritic cells; IFN, interferon; MN, monocytes; N, neutrophils; NK, natural killer cells; T, T lymphocytes; Th, T helper; Treg, regulatory T cells; WBC, white blood cells.

Multiple fluctuations in hormonal exposure occur throughout life, but the greatest changes occur during *in utero* development, puberty, and menopause (Figure 3). *In utero*, maintenance of foeto-maternal immune tolerance is critical to enable full term pregnancy. Fetal and maternal Th1 responses are harmful to the pregnancy and associated with pre-term labor and spontaneous abortion (66). High progesterone levels contribute to polarizing maternal and fetal immunity toward a more tolerant Th2 response (67, 68). The progesterone receptor (PR) is expressed by NK, DC, macrophages, and T cells (69). Activation of PR (a) downregulates TNF, IL-1β, IL-6, and IL-23 from DC (70–72); (b) decreases microbial activity of macrophages as well as downregulating inducible nitric oxide synthase (iNOS) and nitric oxide (NO) production and polarizing macrophages to an M2 alternative-type (73); (c) decreases cytotoxicity and interferon-gamma (IFNγ) production from NK and CD8 cells (62, 74); and (d) increases expression of anti-inflammatory transcription factor FOXP3 in regulatory T cells (Tregs) and reducing IL-17 production (75). The high number of Tregs in the fetal circulation contribute to fetal tolerance of maternal alloantigen. This Th2 bias and increased number of peripheral Tregs persist into the neonatal period and affect responses to foreign antigen (49). This tolerant phenotype, along with the reduced antimicrobial activity and poor antigen presentation of innate immune cells, would be expected to contribute to impaired granulomatous control of intracellular *M. tuberculosis* infection in infants, resulting in high risks of disease progression and extrapulmonary dissemination (Figures 1, 2).

Whilst babies are born with an “underdeveloped” immune system due to low antigen exposure *in utero*, they experience rapid antigen exposure immediately following birth. During this period, both arms of the innate and adaptive immune system mature. Monocyte and DC maturation occurs during the 1st year of life (76), whilst NK cell function normalizes around the age of 5 years (77). NK cell function also changes as children progress into adolescence, switching from IFNγ-producing to more cytotoxic (77, 78). Infants have impaired type I IFN and Th1 (TNF, IFNγ, IL-12) responses and higher Th2/Th17 and IL-10 production (79). Early monocytes and DCs also show less polyfunctionality in cytokine production (80). This underdeveloped innate and adaptive immune system, with suboptimal innate cell-mediated killing and suppressed effector cell responses, is hypothesized to lead to poor outcome following *M. tuberculosis* infection, including widespread dissemination.

Puberty is defined by adrenarche and gonadarche, two temporally correlated but physiologically distinct events. Adrenarche, which begins around age 6, is marked by the onset of production of dehydroepiandrosterone (DHEA), the primary adrenarche hormone, and its storage form, DHEA-sulfate (DHEAS), hereafter collectively referred to as DHEA(S). DHEA(S), which leads to the development of axillary and pubic hair and sweat glands, is the most abundant steroid hormone in the body (81). In gonadarche, production of oestradiol or testosterone signals the maturation of reproductive organs and secondary sex characteristics. In addition, leptin—an adipocyte-produced hormone that regulates energy expenditure and signals the body is prepared for sexual maturation—increases at the onset of puberty (82).

In both sexes, the number of circulating leukocytes changes during the ages of 12–18 years, predominated by a drop in the high number of B cells found in young and primary school children and a rise in helper T lymphocytes (83). The functional requirement of these T cells for TB immunity was demonstrated in a recent study investigating the whole blood transcriptional signature of children and adolescents with pulmonary and extra-pulmonary TB. It identified a decline in T cell transcripts with increasing disease severity, with lower transcript levels found in those with TB meningitis compared to those with pulmonary TB. Moreover, this decline was associated with a functional defect in T cell proliferation following broad T cell receptor stimulation, which recovered after treatment (84).

The lymphocyte predominance seen in pre-pubertal years is overtaken by a predominance of neutrophils during adolescence and into adulthood (83), with neutrophils being considered a driver of cavitation and immunopathology in adult pulmonary TB (85). Early studies also show that the phagocytic capacity of neutrophils, at least to *Staphylococcus aureus*, decreases significantly during adolescence, with maximal kill peaking at 14 years of age (86). A decline in neutrophil phagocytic ability after the age of 14 would thereafter correspond to the rise in TB risk observed in late adolescence. The decreased phagocytic activity may be hypothesized to lead to increased necrosis and more tissue damage synonymous with adult pulmonary TB.

## Impact of Sex on the Host Response to *M. tuberculosis*

### Sex Differences in Inflammatory Responses

**Figure 4:**
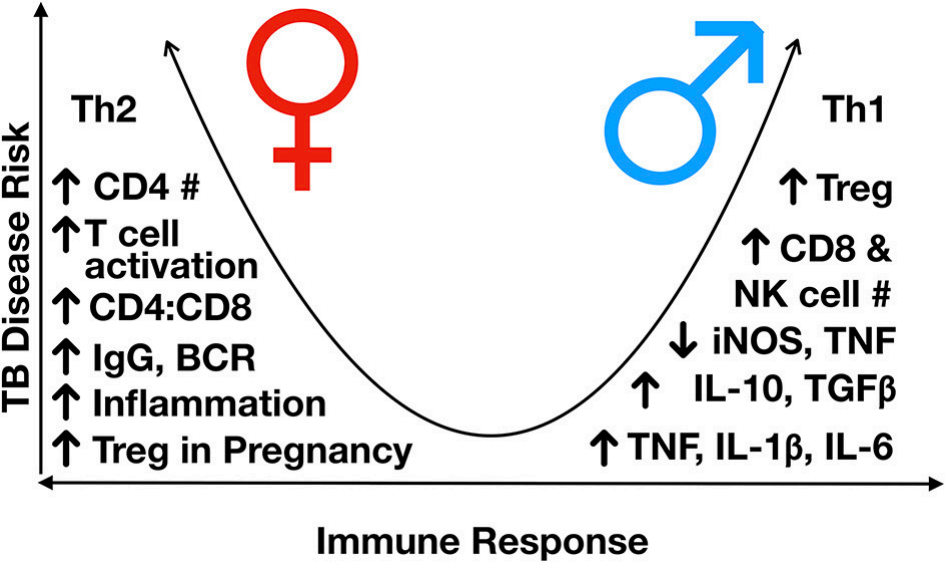
Immunological differences observed between males and females, post-puberty. TB disease risk increases as the immune response more heavily favors either Th2 or Th1 skewing, with a more balanced Th1/Th2 response having the lowest risk of disease progression. In general, males are Th1 skews and females Th2 skewed, although females have a higher inflammatory response to an exogenous stimulus, partly mediated by variable X-inactivation and the presence of estrogen response elements in many immune response genes, leading to higher responses once activated. BCR, B cell receptor; Ig, immunoglobulin; IL, interleukin; NK, natural killer; iNOS, inducible nitric oxide synthase; Th, T helper; TGFß, transforming growth factor-beta; TNF, tumor necrosis factor; #, number.

In general, it is considered that of the sex hormones, oestrogens have immune-enhancing effects, such that the level of inflammatory response is generally higher in females, whilst progesterone and androgens, such as testosterone, exert mainly immunosuppressive properties (Table 2) (87). Moreover, males generally have a Th1 skewed response, and females, Th2 (63). Thus, females are thought to have a stronger innate and adaptive immune response, although overall skewed to a Th2 state (Figure 4) (89).

**Table 2:** Impact of hormones on components of the immune system implicated in the immune response to *Mycobacterium tuberculosis*, as reported in humans, animals, and *in vitro* models.

**General response**	**DHEA(S)**	**Adiponectin**	**Leptin**	**Oestradiol**	**Progesterone**	**Testosterone**
Immune phenotype (87, 88)	Enhancing	Suppressive	Enhancing	Enhancing	Suppressive	Suppressive
T cell polarization (63, 67, 68, 87, 89)	Th1		Th1	Th2	Th2	Th1
**MACROPHAGE ACTIVITY**						
TNF expression^§^ (64, 70–72, 90–97)	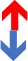	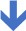	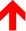	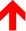 at low levels, 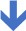 at high levels^#^	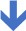	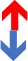
IL-12 expression^§^ (63, 64, 90, 91, 97, 98)	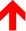	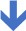	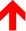	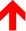	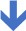	
IL-1β, IL-6 expression (70, 71, 99–101)				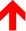 at low levels, 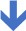 at high levels^#^	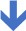	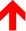
IFNα expression (102)				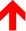		
Foam cell differentiation (103–109)	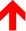	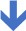	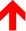	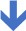	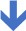	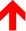
Phagocytic activity (97, 110, 111)			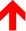	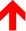	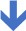	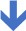
Microbial activity (iNOS, NO) (73, 112–115)			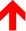	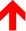	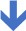	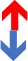
Autophagy of *Mtb*-infected macrophages (116)	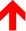					
Lung granuloma formation^§^ (114, 97, 117)			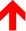	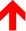		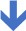
**DENDRITIC CELL ACTIVITY**						
MHC expression (70, 90, 91, 118)	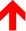	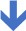		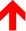	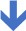	
CD1a^+^ expression (119)		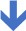				
**NEUTROPHIL ACTIVITY**						
Numbers (62, 120)				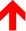		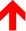
Degranulation (62)				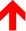		
Apoptosis (121)				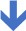	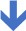	
Phagocytosis (122–124)				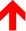		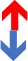
**LYMPHOCYTE ACTIVITY**						
IFNγ expression (63, 64, 90, 91, 97, 117, 125, 126)	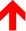	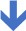	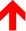	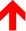	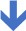	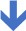
Th1 differentiation (87, 91, 127, 128)	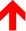		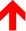	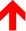 at low levels, 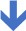 at high levels^#^	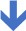	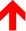
Th2 (63, 67, 68, 87, 89, 129)				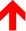	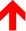	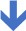
Th17 (75, 87, 130)				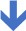	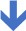	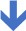
FOXP3, Treg (49, 131, 132)				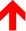	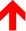	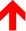
Cytotoxicity of NK and CD8 (62, 74, 98, 133, 134)				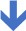	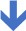	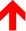
IFNγ expression from NK and CD8 (124, 126, 135)				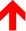	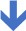	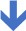
B cell proliferation, Antibody production (87, 89, 134, 136, 137)				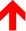	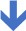	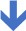

*DHEA(S), dehydroepiandrosterone (sulfate); IFN, interferon; IL, interleukin; MHC, major histocompatibility complex; Mtb, Mycobacterium tuberculosis; NK, natural killer; NOS, nitric oxide synthase; Th, T helper; TNF, tumor necrosis factor; Treg, regulatory T cell. Red arrow indicates higher and blue arrow indicates lower, a blank box indicates no reported significant difference*.

§ *Also expressed by or involves dendritic cells and Th1 cells*.

# *High levels of estrogen occur during the follicular phase of the menstrual cycle and during pregnancy*.

Androgens, including dihydrotestosterone (DHT) and testosterone, suppress Th2/Th17 responses and decrease antibody responses and B cell proliferation (87, 88, 129, 138). With regard to innate cells, testosterone treatment increases IL-12, and IL-1β production in monocytes, whilst reducing IL-6 (99, 100). In DCs it reduces Th2 responses including suppressing IL-4, IL-10, and IL-13 production (129). Conversely, following TLR stimulation, estrogen induces the genes *TLR7, MYD88, RIG1, IRF7, IFNB, AJK2, STAT3, NFKB, IFNG*, and *TNF*—many of which have estrogen response elements in their promoters (62, 139). Consequently, these genes have higher expression in females after vaccination (139). Estrogens also elicit rapid non-nuclear responses via binding estrogen receptors associated with the inner plasma membrane. This activates NO and cyclic AMP production, the mobilization of intracellular calcium and the stimulation of protein kinase pathways, such as PI3K/AKT and ERK (112).

With respect to changes in lymphocyte populations as children progress into adolescence, as sex hormones begin to exert their effects on cell development, differences in the frequency of different cell populations begin to emerge. Males have been shown to have a higher number of circulating CD8 and NK cells, indicating a higher frequency of cytotoxic cells (62, 133). Males develop a more robust innate immune response early in development (140), which may contribute to early infection control and slower disease progression. However, once disease develops, these features may contribute to greater immunopathology and severity of disease in males. Conversely, females develop higher circulating levels of immunoglobulins (Igs) and B cell receptor expression (136); a lower number of B cells; a higher number of CD4+ T cells; and a higher CD4:CD8 ratio (86, 141, 142). Given the recent support of Fc-mediated antibody protection in latently infected individuals, including enhanced phagolysosomal maturation and antimycobacterial activity of macrophages (143), the decreased antibody production and B cell proliferation in males may contribute to gender differences in TB prevalence and presentation.

The difference in TB case prevalence between genders begins to appear in mid-adolescence (144). This timing corresponds to the steep rise in production of adrenarchal and gonadarchal hormones, which decline in the fourth or fifth decade of life (Figure 3) (81, 145–147). Levels of leptin, also follow the same pattern (148). These hormonal declines correspond temporally to the fall in TB rates, which occurs earlier and faster in women and corresponds to the transition into menopause, which generally begins in the late 30s or early 40s (62, 144). The association of a rise and decline in TB risk with the rise and decline in sex hormones supports a potential role for these hormones in controlling the inflammatory imbalance that leads to TB disease progression.

Sex differences in immune responses have been identified for the “type” of inflammatory response produced, i.e., Th1 vs. Th2; the level of regulation governed by transcriptional and post-transcriptional modifications; and differences in the absolute number of different circulating cell populations (Table 2 and Figure 4). These differences can be due to differential exposure to sex hormones, as well as genetic and epigenetic differences impacting gene expression.

As the outcome of infection depends on the immune pathways activated by the pathogen and the pre-existing environment and propensity of the host to respond to those stimuli (140, 149), the sexually distinct activation pathways likely impact infection outcome. It has been hypothesized that even though females elicit more immune-enhancing effects, due to their inherent Th2 skewing, they have a more controlled response to Th1-inducing pathogens (140). Males, although generally immune-suppressive, are Th1-skewed, and thus are likely to elicit an exaggerated response to Th1-inducing pathogens, such a *M. tuberculosis* (140). Conversely, the strong Th2 and humoral response of females is hypothesized to be the underlying cause of higher rates of autoimmune diseases experienced by females (89, 150).

This gender difference in inflammatory response may explain the difference in *Mtb* lineages identified in TB patients due to an interaction between the inherent host inflammatory response and inflammatory pathways activated by different *Mtb* lineages (151). A recent large population study in Vietnam tracing 1635 *Mtb* strains by whole genome sequencing found that young people and females in particular are more susceptible to TB caused by the Beijing 2.2 lineage whilst males and the elderly are more susceptible to Lineage 1 strains (152).

### Genetic and Epigenetic Causes of Differential Sexual Responses to Infection

Differential expression of genes on the X and Y chromosomes defines sexual development. Male development is mediated by the SRY gene on the Y chromosome, whilst females regulate X chromosome expression via X-linked gene inactivation. The differential level of X inactivation that occurs between males and females can impact the level of expression of X-linked genes between sexes [reviewed in Markle and Fish (60)]. The X chromosome also contains ~10% of all 800 microRNA produced by humans, whilst the Y chromosome accounts for only 2% (153). Incomplete X-inactivation in females therefore increases the expression of regulatory miRNA, the expression of which can also be under hormone control, further increasing gene regulation differences between sexes. Toll-like receptor 7 (TLR7) which is activated by viral cytosolic nucleic acids, has higher expression in females than males (102). Subsequently, plasmacytoid dendritic cells (pDC) of females make double the amount of IFNα to TLR7 ligands, such as HIV (154). Type 1 interferons, IFNα and IFNβ, have recently been shown to have detrimental effects on *M. tuberculosis* control depending on their context of activation (155, 156); this finding suggests this difference in females may contribute to exacerbating an inflammatory imbalance, skewed to *M. tuberculosis* survival and proliferation. Conversely, in response to TLR8 and TLR9 agonists, male androgens suppress macrophage phagocytic activity, and correlate to higher IL-10 secretion (157). In addition, males have higher pro-inflammatory production following LPS stimulation of TLR4, including higher TNF production from neutrophils (158). Thus, viral and bacterial responses in males and females may also differ according to TLR expression and activation in each sex. Of particular relevance to TB pathology, compared to monocytes and T cells, neutrophils have greater differences in global methylation and gene expression profiles between males and females (159). Twenty-one genes expressed from neutrophils were identified to be differentially expressed between sexes. Meanwhile, one of the top two differentially expressed genes between male and female neutrophils, SEPT4, is often found in the neutrophil-driven whole blood signatures of TB (160–162).

### Sex Hormone Impact on Response to *M. tuberculosis*

As shown in Table 2, hormones may impact adolescent TB through multiple plausible mechanisms and potentially in a multifactorial manner. Increased DHEA(S) and leptin levels may promote foam cell differentiation (103–106), leading to disease progression and lung cavitation. DHEA(S), estradiol, and leptin may contribute to disease severity by promoting aggressive Th1 responses (63, 90, 91, 118, 127, 128). A study comparing TST response after Bacillus Calmette-Guérin (BCG) vaccination gives clinical support to the association between DHEA(S) and Th1 responses; in this study, pubertal subjects had higher DHEAS levels and larger TST reactions than pre-pubertal subjects (163). Likewise, the link between gonadarche and disease severity is strengthened by a guinea pig model that demonstrated associations between increased exposure to oestradiol and testosterone and greater mortality and more extensive, caseous lung lesions (164). Suppression of TNF by DHEA(S) may also contribute to disease progression (92–96).

The greater resistance of female mice to mycobacterium species has been demonstrated in a number of studies (113, 114, 165, 166). In a C57BL/6 mouse model of TB, male mice have accelerated disease and increased mortality compared to females. The male mice had increased lung bacterial loads, increased iNOS, IFNγ, IL-1α/β, and IL-6 production early and late in infection, and higher levels of various inflammatory chemokines, all of which correlated with increased bacterial burden (114). Castration of male BALB/c mice reduced mortality and increased levels of inflammation to those seen in female mice (113). However, converse to what was seen in C57BL/6 mice, during early infection, female and castrated BALB/c expressed higher lungs levels of *TNF, IFNG, IL12, iNOS*, and *IL17* than non-castrated males. Moreover, castration 60 days after infection increased the survival of male mice, decreasing bacterial load and increasing *TNF, IFNG*, and *IL12*. Interestingly, whilst female castration resulted in declines in bacterial numbers early during infection, they rose at day 60 compared to non-castrated females. Testosterone treatment of female mice also increases susceptibility to *M. marinum* (165), whilst oestradiol treatment of ovariectomised mice increases mycobacterial killing, synergistically with IFNγ (167). Testosterone treatment of rats increased recruitment of inefficient neutrophils following LPS treatment, with impaired bacterial activity decreased MPO activity and increased IL-10 and TGFβ expression (168). Together, these studies suggest that high levels of testosterone can contribute to disease progression in males, whilst estrogen elicits a more protective response. However, the difference in genetic backgrounds of the animals infected also creates different inflammatory processes correlated to disease. Thus, the effect of hormones will be governed by the overall inflammatory state of the individual, which may be further governed genetically, epigenetically or environmentally by other co-morbidities, including co-infections.

## Beyond Age- and Sex-Related Immunological Changes in Children and Adolescents

Although age- and sex-related changes in the immune response to *M. tuberculosis* are a critical factor in the relative reduction in TB disease prevalence in primary school children and increase in adolescence, it is important to consider other contributing factors (Figure 3). The degree of TB exposure is one alternative explanation for and/or contributing factor to different risks of disease progression. A study from Canada found that among recently infected individuals, those who developed TB disease had exposure to a higher number of infectious TB cases than those who did not (169). At the same time, a recent animal study suggested that repetitive aerosol exposure with *M. tuberculosis* drives greater lung tissue destruction, including cavitation, than a single exposure (170). This explanation fits with the increased socialization in adolescence that is assumed to occur in many societies, and may contribute to increased risk of TB progression (171). This generalization about adolescent social mixing is supported by data from various settings, including eight European countries, rural Andean communities in Peru, a South African township, and a city in Siberia, Russia. These cross-sectional surveys all found that on a daily basis, adolescents come into contact with 1.5- to 3-times as many individuals as young children do (172–176). However, the hypothesis that a higher number of TB exposures increases the probability of TB disease progression does not account for the higher risks observed in young children vis-à-vis primary school children, or adolescent girls vis-à-vis adolescent boys. Young children come into contact with fewer individuals on a daily basis than primary school children (172–176), and to our knowledge, there are no data to suggest that adolescent girls have more frequent exposure to infectious TB cases compared to adolescent boys. The social mixing studies showed no difference in mean number of contacts for females vs. males, but these data were not age-disaggregated (172, 175).

On the other hand, if one of the drivers of risk of disease progression is the intensity of exposure (i.e., the size of the bacillary load), then the increased risk during early childhood makes more sense. Data from various settings support the idea that young children, particularly those <2 years of age, spend more time with household contacts than primary school children and adolescents (173, 175, 176); thus, they are exposed to a larger inoculum of *M. tuberculosis* from caregivers with TB disease. For this reason, young children are more likely than older children and adolescents to become TB-infected from a household exposure (177, 178). However, when comparing primary school children and adolescents, social mixing studies did not find significant differences in the amount of time spent with individual contacts (173–175).

Initiation of substance use and sexual activity also may contribute to the increase in TB disease progression and mortality in adolescents. Recreational drugs, alcohol, and smoking all are associated with increased risk of TB disease (179–182). Sexual activity may lead to pregnancy, which can predispose a woman to TB disease progression due to a wide range of immunosuppressive effects (183, 184). Moreover, sexual activity increases the risk of acquiring transmissible infections, including HIV.

Among co-infections that may contribute to the age- and sex-related changes seen in TB risk, HIV is likely the most significant. As individuals enter adolescence and commence sexual activity, girls are more likely than boys to acquire HIV (185). Globally, adolescent females have higher HIV seroprevalence than adolescent males (186). Because HIV dramatically increases the risk of TB disease progression following infection (187, 188), this difference in seroprevalence may contribute to the higher TB disease risk in adolescent girls. It is possible that HIV prevalence is lower in the primary school years compared to both early childhood and adolescence, since untreated vertically transmitted HIV leads to death in ~60% of children before age 2 years (189) and primary school children have minimal risk of horizontal transmission. However, this possibility is difficult to confirm because, to our knowledge, global childhood HIV prevalence has not been disaggregated into 5-year age bands (190). The data on age-based differences in risk of TB disease progression were collected prior to the HIV epidemic and exist in regions with little HIV; thus, HIV epidemiology cannot be the sole reason for age- and sex-based discrepancies in TB risk.

Other pathogens also may alter an individual's susceptibility to TB disease. Cytomegalovirus (CMV) has been implicated in TB disease pathogenesis (191); it exerts profound immunodysfunction on infants, and has been associated with increased TB risk in this age group (192, 193). Moreover, increases in CMV seroprevalence are highest during infancy and adolescence, and the acceleration of CMV seroprevalence is steeper in adolescent girls than boys (191).

TB epidemics demonstrate a seasonal pattern and follow influenza outbreaks (194–196). A study conducted in a Danish TB sanatorium in the mid-twentieth century found that a strong association between influenza and clinical exacerbation of TB (197). Other studies have found associations between influenza and excess TB mortality (198–200). It is possible that influenza—and perhaps other viruses affecting the lower respiratory tract—may predispose to TB disease progression and mortality by disrupting mucosal integrity and altering host immunology. This link could partially explain the increased TB risks experienced by young children, who are vulnerable to severe lower respiratory disease due to influenza and other respiratory viruses. As children reach primary school age, they become less vulnerable to lung disease from common respiratory viruses (201). With the notable exception of the 1918–1919 influenza pandemic, adolescents do not experience increased morbidity and mortality from respiratory viruses (200). Therefore, this hypothesis does not explain the rise in TB risk during adolescence.

Finally, helminths and malaria infections are both common in young children in high TB-burden settings and cause immune dysregulation that could impact TB risk (202–205). However, the incidence of these infections do not rise again during adolescence, so they do not explain the adolescents' elevated TB risk. It is therefore possible that different etiologies contribute to the elevated risk in young children and adolescents. Further research is needed to disentangle the contribution of exposure intensity and frequency, substance use, and co-infections to the U-shaped pattern of TB risk during childhood and adolescence.

## Implications

The Wonder Years offer a unique insight into the immunological protection against TB disease progression. We have made the case that if infected during this period the host response is effective in containing *M. tuberculosis* within granuloma in contrast to younger children, who more commonly progress to disseminated disease. In addition, primary school children are also less likely to mount a tissue damaging, inflammatory response to *M. tuberculosis*. They frequently experience localized bacillary replication within the lung and are less likely than post-pubescent adolescents and young adults to develop cavitation. Thus, comparing age-related immunological changes that occur before, during, and after puberty may reveal immunological pathways that could be targeted to promote balanced protective immunity.

Development of an effective TB vaccine has been hampered by insufficient understanding of protective immunity. Given that the presentation of disease and underlying immunological responses change during different risk periods, the approach to inducing an optimal vaccine response in young children may differ to the approach needed in adolescents and adults. Strategies in infants or young children could be aimed at either preventing the establishment of infection by priming the innate immune system or promoting cell-mediated immunity that provides superior protection from dissemination than BCG. Strategies for adolescents could aim to: clear mycobacteria before the stage of increased progression risk; enhance containment within the granuloma; or attenuate the excessive inflammatory response should containment break down. It may be appropriate to revaccinate children prior to adolescence (potentially with a vaccine of different mechanism) or provide host-directed therapies following exposure. Furthermore, the difference in host response of adolescent boys and girls may need to be modulated by vaccines and immunotherapies in different ways. This is supported by the first successful outcome of the new M72 TB vaccine, which demonstrated 57% efficacy in the population, but 75% efficacy in males and 84% efficacy in those ≤ 25 years, although this secondary analysis is confounded by the enrolled population being skewed toward young males (206). Future vaccine studies must be designed with sufficient power to rigorously test the effect of age and gender on trial outcomes.

In addition, approaches to diagnostics may have to factor in age and sex. Recently, there has been great interest in whole blood transcriptional signatures as potential diagnostics and prognostics for TB infection and disease. These biomarkers identify a characteristic host response to a particular disease process; however, in TB, pathogenesis of disease may vary by age and, to a lesser extent, by sex. As a result, a transcriptional signature that performs well in diagnosing adult-type pulmonary disease may not capture disease in children, and vice versa.

Ultimately, more research is required to better understand how immunological responses to *M. tuberculosis* change with age and sex. Animal studies, including the use of juvenile animals, could assist in delineating the impact of age and sex on host response to *M. tuberculosis*, as well as the changing disease phenotypes that result. It would also be possible to evaluate different vaccination strategies and host-directed therapies to prevent infection and/or disease progression in these animal models. Longitudinal cohorts of young children either prior to TB exposure, or following known exposure, could contribute vital information. In these cohorts, blood samples could be taken at regular intervals to document cell phenotypes and functions and to understand immunological risk factors for disseminated disease. For adolescents, longitudinal cohorts would also be informative, with children identified prior to puberty and with an evaluation of how host immunological responses change with age, puberty, and with sex. Specific attention to methylation profiles of boys and girls at different ages may identify the changes induced by puberty which could impact disease risk and protective immunity. Impact of co-infections in both young children and adolescents would be important to consider. Although longitudinal cohorts are expensive to conduct, it is often strategic and efficient to undertake such studies within the framework of therapeutic clinical trials or vaccine trials.

## Conclusions

Primary school children teach us that there is still much about TB that we do not understand. They sit within the flexion point where TB pathology and risk changes; the immunological changes that occur during early childhood and puberty are likely to impact the response to infection and risk of disease. The difference in TB risk that emerges after puberty also indicates that diagnostics and strategies for prevention and treatment may need to be targeted according age and sex.

## Author Contributions

All authors listed have made a substantial, direct and intellectual contribution to the work, and approved it for publication.

### Conflict of Interest Statement

The authors declare that the research was conducted in the absence of any commercial or financial relationships that could be construed as a potential conflict of interest. The handling editor is currently co-organizing a Research Topic with one of the authors AC, and confirms the absence of any other collaboration. The reviewer SG declared a shared affiliation, though no other collaboration, with one of the authors SC to the handling Editor.
